# Functional Abnormality Associated With Tau Deposition in Alzheimer’s Disease – A Hybrid Positron Emission Tomography/MRI Study

**DOI:** 10.3389/fnagi.2021.758053

**Published:** 2021-10-13

**Authors:** Liping Fu, Zhi Zhou, Linwen Liu, Jinming Zhang, Hengge Xie, Xiaojun Zhang, Mingwei Zhu, Ruimin Wang

**Affiliations:** ^1^Department of Nuclear Medicine, China-Japan Friendship Hospital, Beijing, China; ^2^Department of Neurology, China-Japan Friendship Hospital, Beijing, China; ^3^Medical Research Center, Peking Union Medical College Hospital, Chinese Academy of Medical Sciences and Peking Union Medical College, Beijing, China; ^4^Department of Nuclear Medicine, The First Medical Center, Chinese PLA General Hospital, Beijing, China; ^5^Department of Neurology, The Second Medical Center, Chinese PLA General Hospital, Beijing, China

**Keywords:** Alzheimer’s disease, PET/MRI, tau, fMRI, functional connectivity

## Abstract

**Objective:** To investigate the characteristics of tau deposition and its impact on functional connectivity (FC) in Alzheimer’s disease (AD).

**Methods:** Hybrid PET/MRI scans with [^18^F]-THK5317 and neuropsychological assessments were undertaken in 26 participants with AD and 19 healthy controls (HC). The standardized uptake value ratio (SUVR) of [^18^F]-THK5317 PET imaging was compared between the AD and HC groups. Significant clusters that revealed higher tau deposition in the AD group compared to the HC group were selected as regions of interest (ROI) for FC analysis. We evaluated the difference in the FC between the two groups for each ROI pair. The clinical and radiological characteristics were compared between the AD patients with negative FC and AD patients with positive FC for exploratory analysis.

**Results:** The bilateral inferior lateral temporal lobe, dorsal prefrontal cortex, precuneus, posterior cingulate cortex, hippocampus, and occipital lobe showed significantly higher [^18^F]-THK5317 accumulation in AD patients. Decreased FC in regions with higher SUVR was observed in AD patients, and the FC strength was negatively correlated with regional SUVR. Patients with a positive FC exhibited older ages, better cognitive performances, and a lower SUVR than patients with a negative FC.

**Conclusions:** An impact of tau deposition was observed on FC at the individual level in AD patients. Our findings suggested that the combination of tau-PET and rs-fMRI might help predict AD progression.

## Introduction

Abnormal deposition of amyloid-β (Aβ) and tau is the hallmark of pathology in Alzheimer’s disease (AD). According to the pathological classification model, the accumulation of Aβ plaque (A) deposition occurs first, followed by phosphorylated tau (T) deposition and downstream neurodegenerative (N) events (Jack et al., 2018).

Tau protein plays an important role in promoting the stability of tubulin assemblies and maintaining the microarchitecture of neurons (Kametani and Hasegawa, 2018). Recent advances in molecular neuroimaging, including positron emission tomography (PET), have enabled the identification and quantification of pathological proteins *in vivo*. PET with tracers, including [^18^F]-THK5317 and [^18^F]-AV1451, is a well-established neuroimaging technique for measuring regional tau burden. Previous studies with tau-PET demonstrated that the spatial pattern of tau deposition was similar to the key features of the Braak histopathological stages illustrated by autopsy-based assessments (Schwarz et al., 2016). Compared with Aβ, the spread of tau pathology shows a strong relationship with the progression of AD (Kametani and Hasegawa, 2018).

The deposition of pathological proteins contributes to the cascade of functional and morphological changes in the brain. There are extensive reports indicating that tau causes direct toxic effects on neuronal activity and synaptic plasticity in AD, leading to disruption of functional connectivity (FC), which assesses the correlation between spontaneous activity fluctuations in remote brain regions (Busche et al., 2019). The combination of tau-PET and multi-modal MRI facilitates *in vivo* investigation. Recently, hybrid PET/MRI has become available in clinical practice, which provides the opportunity to combine PET and MRI in a single imaging session. Neuroimaging studies using resting state functional MRI (rs-fMRI) and tau-PET support the idea that tau disrupts FC in AD by showing that tau accumulation weakens FC.

Previous work primarily focused on FC disruption in known brain functional networks. Based on these observations, recent studies illustrated that highly connected nodes, which also are called hubs, displayed more tau pathology, and increases in tau burden were associated with decreases in FC at these same nodes (Cope et al., 2018; Yokoi et al., 2018; Franzmeier et al., 2019, 2020). These results provide evidence for the “network degeneration hypothesis,” in which the spreading of pathological tau propagates *trans-*neuronally in a prion-like manner (Kametani and Hasegawa, 2018). However, these studies examined the alterations in FC and tau deposition separately. Therefore, the direct interaction of pathological tau deposition and regional FC and the influence on cognitive impairment remains unclear. In this study, using hybrid PET/MRI with [^18^F]-THK5317, we investigated the characteristics of tau deposition and its impact on FC in AD patients at the individual level.

## Materials and Methods

### Participants

Twenty-six AD and 19 HC subjects were recruited at the Chinese PLA General Hospital. The clinical diagnosis of AD was made based on the criteria for dementia cited in the International Classification of Diseases, 10th Revision (ICD-10) and the criteria for probable AD from the National Institute of Neurological and Communicative Disorders and the Stroke/Alzheimer Disease and Related Disorders Association (NINCDS-ADRDA) (McKhann et al., 2011). All participants were clinically assessed using the Clinical Dementia Rating (CDR) (Morris, 1993), which categorized the participants as HCs (CDR = 0) or patients in the early stages of AD (CDR>0.5). The inclusion criteria for AD included: (1) significant episodic memory problems reported by the patient, a relative, or caregiver; (2) impaired performance on a general cognition test [Mini-Mental State Examination (MMSE) score < 24] and in activities of daily living (ADL); (3) medial temporal lobe atrophy as assessed with the visual atrophy rating scale (Scheltens et al., 1992). Also, AD patients exhibited positive results obtained with an amyloid PET [(^11^C)-PIB] scan. Participants were excluded if they presented one of the following criteria: (1) structural abnormalities that could result in dementia, including cortical infarction, tumors, or a subdural hematoma; (2) concurrent illnesses other than dementia that interfered with cognitive function at the time of the MRI examination; (3) metabolic conditions such as hypothyroidism, and vitamin B12 or folic acid deficiencies. The HCs had no history of neurological or psychiatric disorders, sensorimotor impairment, or cognitive complaints, no abnormal anatomical findings based on conventional brain MRI, and no evidence of cognitive deficits on neuropsychological tests. All participants exhibited right-hand dominance. Written informed consent was obtained from each participant, and this study was approved by the institutional review board of the Chinese PLA General Hospital. We conducted this study in compliance with the principles of the Declaration of Helsinki.

### Positron Emission Tomography/MRI Scans

Forty minutes after the injection of approximately 370MBq (4.44–5.55 MBq/kg) of [^18^F]-THK5317, each participant was positioned in a hybrid PET/MRI system (Biograph mMR, Siemens Healthineers, Erlangen, Germany) that consisted of a whole-body PET and a 3.0-T MRI. This hybrid scanner enables the acquisition of 127 transaxial planes over a 25.8 cm axial field of view, which allows the entire brain to be imaged in a one-bed position. The [^18^F]-THK5317 PET scan started 40 min after the tracer was injected and lasted for 20 min. It was carried out simultaneously with the MRI scan, which included attenuation correction acquisition (ultra-short echo time (UTE) sequence, TE1/TE2 = 0.07/2.46 ms, TR = 11.94 ms, flip angle 10°, 192 slices, matrix size: 192 × 192 × 192, FOV = 300 mm × 300 mm, voxel size 1.6 mm × 1.6 mm × 1.6 mm, acquisition time 1:40 min/bed position), high-resolution sagittal 3D T1-weighted magnetization-prepared rapid gradient echo (MPRAGE) acquisition (TR = 1,900 ms, TE = 2.44 ms, inversion time = 900 ms, slice thickness = 1 mm, matrix = 256 × 256, FOV = 250 mm × 250 mm, voxel size 1.0 mm × 1.0 mm × 1.0 mm, acquisition time 8:54 min/bed position), a transverse T2-weighted turbo spin echo acquisition (TR = 4,500 ms, TE = 85 ms, flip angle 150°, 25 slices, slice thickness 4 mm, FOV = 220 mm × 220 mm, voxel size 0.7 mm × 0.7 mm × 4.0 mm, acquisition time 2:32 min/bed position) and single shot gradient echo-planner imaging with blood oxygenation level dependence (BOLD, TR = 2,000 ms, TE = 30 ms, slice thickness = 3.5 mm, slice = 43, Matrix = 64 × 64, FOV = 224 mm × 224 mm, acquisition time 8:00 min/bed position). The 20 min (taken at 40–60 min) of PET data were converted into standardized uptake value (SUV) images for further analysis using an ordered subset expectation maximization algorithm with settings of iterate = 3, subset = 21, matrix = 336 × 336, and a Gaussian filter of 4 mm in full-width half-maximum (FWHM). All subjects underwent THK5317-PET and 3D-T1WI MRI scanning; 22 AD and 12 HC subjects had resting-state BOLD data that were acquired.

All participants underwent a 20-min [^11^C]-PIB PET/MRI static scan, which was performed 40 min after injection (40–60 min) of 4.5 MBq/kg (McNamee et al., 2009). The [^11^C]-PIB was synthesized from its corresponding precursors as described previously (Philippe et al., 2011), with a radiochemical purity of more than 95% and specific activity of 50 GBq/moL (1.48 Ci/moL). The [^11^C]-PIB PET/MRI scan protocol was the same as the [^18^F]-THK5317 PET/MRI scan.

### Radiosynthesis of [^18^F]-THK5317

[^18^F]-THK5317 was prepared using ^18^F- nucleophilic substitution of the tosylate in the precursor in the presence of K2CO3 and Kryptofix-222 using the automatic synthesis module (PET-MF-2V-IT-1, Beijing, China). After radiofluorination, the tetrahydropyranyl (THP) protecting group was removed using HCl (1 M) hydrolysis in the same reaction vessel, and the radiotracer was purified by semi-preparative HPLC. The identity of the radiotracer was confirmed using HPLC co-injection analysis. The radiochemical purity of [^18^F]-THK5317 was greater than 95%, and its specific activities ranged from 90 to 123.5 GBq/μmol, which was corrected at the end of the synthesis.

### Positron Emission Tomography Data Preprocessing

The MRI data were co-registered to the PET [(^18^F)-THK5317 and (^11^C)-PIB] data and spatially normalized to a customized template in Montreal Neurological Institute (MNI) space, which was constructed from the MRI T1W images acquired in this study. The transformative deformation fields were applied to the corresponding PET data, and then isotropic 2 mm spatial resolution-normalized PET data in MNI space were generated. The mean value of the cerebellar gray matter region was used to normalize the intensity values of the PET images, voxel-by-voxel. A 6 mm FWHM Gaussian kernel was used to smooth the normalized PET data. The cut-off value for PIB-PET was 1.4 (Tanaka et al., 2020).

### Resting-State fMRI Data Preprocessing

The resting-state fMRI data were preprocessed using SPM12 software^1^. The first six volumes were discarded to allow for magnetization equilibrium and participants’ adaption to the environment. The slice timing and rigid-body head movement during scans were corrected, so that all images were realigned to the first volume. The excessive motion was defined as a maximum displacement of 3 mm and a maximum angular motion of 3° in any direction. The structural images were co-registered to the first volume of the corresponding functional images, and then segmented into gray matter, white matter, and cerebrospinal fluid. All fMRI images were normalized to the MNI space following motion correction using a diffeomorphic non-linear registration algorithm (DARTEL) (Ashburner, 2007) and resampled to a 2-mm isotropic voxel. The normalized fMRI images were smoothed using a Gaussian kernel of 6 mm FWHM. The fMRI images were finally filtered with a temporal band-path of 0.01–0.1 Hz, and white matter and CSF signals were regressed out.

### Statistics

Data were analyzed using SPSS, version 23.0 (IBM Corp., Chicago, IL, United States). Demographic and clinical variables were assessed for normality of distribution using Kolmogorov–Smirnov tests. Variables exhibiting a normal distribution were compared using the Student’s *t*-test. Gender was analyzed using a Chi-square test. A *p*-value of <0.05 was considered significant.

A voxel-wise two-sample *t*-test was used to compare the difference of the [^18^F]-THK5317 cortical-to-cerebellum standardized uptake value ratio (SUVR) between the AD and HC groups. The false discovery rate (FDR) for multiple comparisons was utilized to control the expected proportion of false-positive results among the suprathreshold voxels with a *p* < 0.05 and a cluster size larger than 800 mm3. Significant group differences (*p* < 0.05) were used as ROIs for rs-fMRI network construction.

Seven regions of interest (ROIs) were extracted from brain regions where tau deposition was significantly increased in the AD group compared to the HC group (details are presented in the “Results” section). An FC analysis was performed among the given ROIs, which was measured using Pearson’s correlation coefficient. The correlation coefficient was derived between the mean time series of each pair of the seven ROIs, with each subject as the FC. A two-sample *t*-test was conducted to compare the FC difference between the two groups for each ROI pair. A significance level of an uncorrected *p* < 0.05 was obtained for the FC comparisons.

## Results

### Demographic Characteristics

Table 1 lists the clinical and neuropsychological data. No significant differences were observed in age, gender, and education level. All AD participants had positive results from the PIB-PET scans. Also, the AD group exhibited significantly lower scores than the HC group for the MMSE.

**TABLE 1:** Clinical and demographic characteristics of all subjects.

	**HCs**	**AD patients**	***p* value**
N	19	26	−
Age (Y)	65.59 ± 8.05	70.68 ± 12.21	0.10
Gender (M/F)	9/8	17/12	0.29
Education (Y)	10.73 ± 4.99	11.23 ± 3.48	0.421
MMSE*	29.33 ± 0.18	20.27 ± 4.69	<0.001

*MMSE scores for AD patients vs. HCs were significantly different by a two-sample *t*-test, *p* < 0.05. Data are mean ± SD or numbers of subjects.

### Voxel-Based Morphometry Analysis of Tau Deposition

**FIGURE 1:**
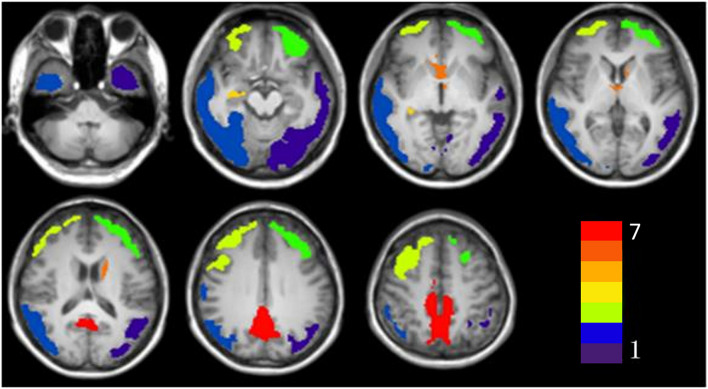
The significant clusters of tau accumulation in Alzheimer’s disease patients. The color bar indicates the different clusters (Cluster 1 to Cluster 7).

The results of the voxel-based morphometry (VBM) analysis of the SUVR maps are shown in Table 2 and Figure 1. The AD group exhibited a significantly higher SUVR than the HC group in numerous cortical and subcortical areas, including the bilateral posterior cingulate cortex (PCC), ventromedial prefrontal cortex, temporal cortex, and parietal cortex (Table 2 and Figure 1). No region was observed that had a significantly lower SUVR in the AD group compared to the HC group.

**TABLE 2:** Significant clusters of higher tau accumulation in Alzheimer’s disease.

**No**	**Cluster voxels**	**Lateralization**	**Regions**	***T***	**MNI coordinate**		
					**X**	**Y**	**Z**
1	8,795	Left	Middle and inferior temporal cortex, Middle and inferior occipital cortex, Angular cortex, Precuneus cortex, Parahippocampal cortex	−7.827	−22	−74	44
2	10,995	Right	Temporal cortex, Middle and inferior occipital cortex, Angular cortex, Precuneus cortex, Parahippocampal cortex	−7.821	52	−60	−18
3	4,791	Left	Dorsal lateral prefrontal cortex	−7.015	−36	32	34
4	4,986	Right	Dorsal lateral prefrontal cortex	−8.390	22	16	60
5	105	Right	Parahippocampus	−4.661	20	−14	−16
6	479	Left	Caudate	−6.383	0	18	−4
7	4,401	Bilateral	Middle and posterior cingulate cortex	−8.087	8	−34	44

### Functional Connectivity Analysis in Regions of Tau Deposition

**FIGURE 2:**
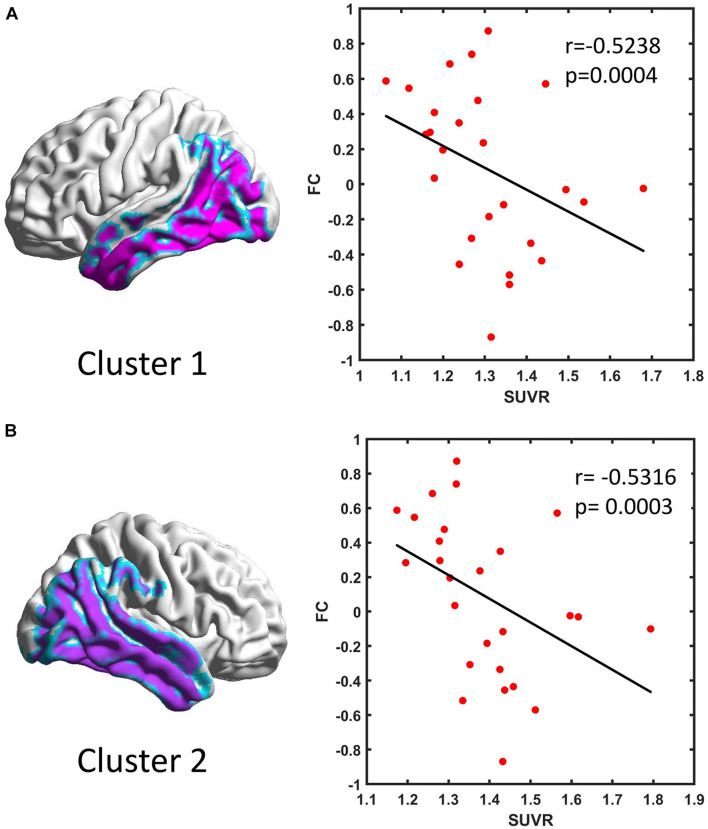
Scatter plot of the functional connectivity and standardized uptake value ratio (SUVR) in Cluster 1 **(A)** and Cluster 2 **(B)** in Alzheimer’s disease patients.

Seven clusters were extracted from the VBM results and used as the ROIs in the FC analysis. The FC between each two ROIs was calculated as the correlation between their time series for all subjects. Compared with the HC group, the AD group showed a significantly decreased connectivity pattern between Clusters 1 and 2 (Table 3). We also found a negative correlation between the FC and SUVR between Clusters 1 and 2 (Figure 2).

**TABLE 3:** Group differences of the FC for each ROI pair between the AD and HC groups.

***T***						
***P***	**Cluster 2**	**Cluster 3**	**Cluster 4**	**Cluster 5**	**Cluster 6**	**Cluster 7**
Cluster 1	2.7241 *0.0096*	−1.5988 *0.1179*	1.0063 *0.3205*	1.3013 *0.2008*	−1.3574 *0.1825*	1.1228 *0.2684*
Cluster 2	–	0.5164 *0.6085*	−1.9831 *0.0544*	−0.8614 *0.3943*	−0.7879 *0.4355*	0.9712 *0.3374*
Cluster 3	–	–	1.0941 *0.2806*	0.3907 *0.6981*	0.4164 *0.6794*	0.1187 *0.9062*
Cluster 4	–	–	–	−0.6602 *0.5130*	0.1553 *0.8774*	−0.4688 *0.6418*
Cluster 5	–	–	–	–	0.4499 *0.6552*	−0.8712 *0.3890*
Cluster 6	–	–	–	–	–	−1.3901 *0.1724*

*In each cell, the upper number is the T value, and the lower number is the *p*-value for each ROI pair.*

### Exploratory Analysis: Clinical and Radiological Characteristics of the Functional Connectivity-Positive and Functional Connectivity-Negative Alzheimer’s Disease Subgroups

**FIGURE 3:**
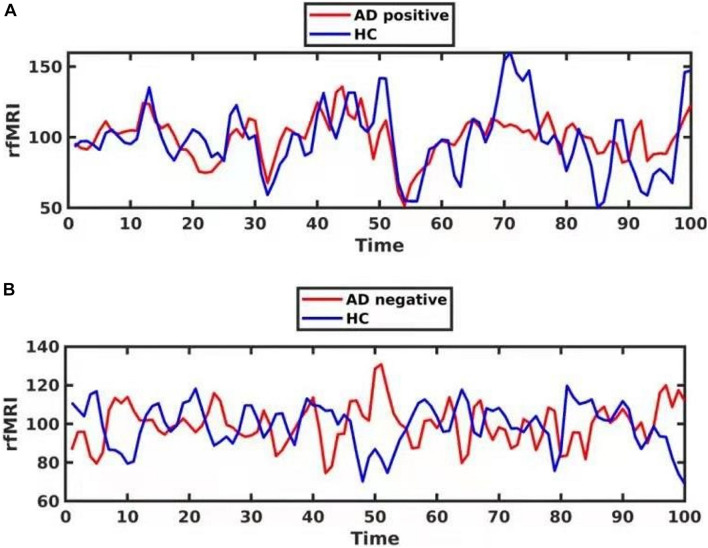
The phase delay of the functional BOLD series for one Alzheimer’s disease (AD) patient with positive functional connectivity (FC) [**(A)**, red] and another AD patient with negative FC [**(B)**, red]. The FC is between Cluster 1 and Cluster 2. The time series for one healthy control is shown in blue.

In the exploratory analysis, we observed that 12 subjects in the AD group showed a negative FC between Clusters 1 and 2. This result indicated that the correlation of paired BOLD signals was negatively correlated, while the other 14 subjects in the AD group showed a positive FC between Clusters 1 and 2. However, all subjects in the HC group exhibited a positive FC between Clusters 1 and 2, which indicated that the correlation was positive or synergic (Figure 3 and Supplementary Material 1).

Based on the FC between Clusters 1 and 2, we divided the AD group into FC-positive and FC-negative subgroups. Table 4 illustrates the clinical and radiological characteristics of these two AD subgroups. When compared to the FC-positive patients, the FC-negative patients were younger and exhibited lower MMSE scores. No significant difference of GMV in Clusters 1 and 2 was detected in the FC-negative and FC-positive subgroups. However, the FC-negative group had greater [^18^F]-THK5317 binding in Clusters 1 and 2.

**TABLE 4:** Demographic and radiological characteristics of the FC-positive and FC-negative groups in AD patients.

		**FC-negative AD**	**FC-positive AD**	***Z* score**	***p* value**
*n*		12	14		
Age*		66.08 ± 9.79	74.71 ± 12.32	−2.04	0.044
Gender (M/F)		4:8	7:7		0.46
MMSE*		17.00 ± 5.31	22.71 ± 2.84	−2.51	0.012
Cluster 1	GMV	5,687.31 ± 1,050.03	6,120.13 ± 920.18	−0.93	0.374
	SUVR*	1.40 ± 0.13	1.22 ± 0.95	−3.45	0.0005
Cluster 2	GMV	6,981.53 ± 1,250.72	7,570.47 ± 1,135.87	−1.18	0.237
	SUVR*	1.48 ± 0.13	1.31 ± 0.10	−3.55	0.0009

*Age, MMSE scores, and SUVR in cluster 1 and cluster 2 for FC-negative AD vs. FC-positive AD patients were significantly different based on a two-sample *t*-test, *p* < 0.05. Data are means ± SD or numbers of subjects.

## Discussion

The main result of this cross-sectional study was the presence of higher tau deposition in the AD group in the bilateral inferior lateral temporal lobe, dorsal prefrontal cortex, precuneus, posterior cingulate cortex, hippocampus, and occipital lobe. FC analysis revealed decreased FC in regions with higher [^18^F]-THK5317 signals. The FC strength was negatively correlated with the regional SUVR in patients with AD. Exploratory analysis revealed that patients with a positive FC were older and exhibited better cognitive performance than patients with a negative FC. In addition, tau accumulation was higher in patients with a negative FC than those with a positive FC. Taken together, these findings suggested that tau accumulation impacted the function and clinical performance of AD patients.

Pathological proteins, including tau, either directly or indirectly, interfere with cerebral function and morphology in AD. Previous studies have demonstrated disruptions of large-scale brain networks in AD, including the default-mode network (DMN) and other networks. However, little is known about alterations in FC patterns associated with high tau deposition. Consistent with previous studies, we demonstrated that regions of tau accumulation were located in important hubs of the posterior DMN, including the precuneus and angular cortex (Franzmeier et al., 2019). With respect to cognitive function, episodic memory is the most vulnerable cognitive subdomain in early AD and relies on the interaction between the DMN and the medial temporal lobe (Ward et al., 2015). Executive function also declined in concert with memory in the early stage of AD, implicating an interplay between the DMN and other relevant networks (La Corte et al., 2016). In AD, where tau accumulation predominates, the affected nodes become weakly connected, which reduces the local efficiency of information transfer.

Negative FC refers to a negative Pearson correlation coefficient for the spontaneous BOLD signal in two brain regions, indicating a negative correlation for the two regions. The origin and interpretation of a negative FC have been debated. Some studies have reported that a negative FC was an artifact induced by the global signal regression and excluded results that included a negative FC to avoid uncertainty (Weissenbacher et al., 2009). However, other studies found that a negative FC could exist without a global signal regression, and the characteristics of a negatively correlated network were not related to the global signal removal (Chang and Glover, 2009; Fox et al., 2009). Additional studies have revealed that a negative FC was associated with predominantly long-range connections, which provides a possible explanation for the underlying neurobiological mechanism. Moreover, Chen et al. (2011) suggested that a negative FC might induce a phase delay in the synchronous signals along the shortest path in the brain functional networks. Because the mechanisms of negative FC still are not well understood (Chen et al., 2011), we compared the cognitive performance between the FC-positive and FC-negative AD subgroups. We observed that AD patients with a positive FC in the tau accumulation region performed better in the cognitive test. Also, the SUVR for [^18^F]-THK5317 was higher in patients with a negative FC. It is worth mentioning that a negative FC was only found in the AD group. These findings support the biological mechanism of negative FC.

In this study, compared with the negative FC subgroup, the positive FC subgroup might be protective to compensate for the neuron injury caused by tau deposition and allows the cognitive performance to be maintained. This mechanism might lead to a period of hyperactivity until the neuronal loss overcomes the compensatory mechanism. However, whether the reserve capacity is related to the cognitive benefit in the presence of severe tau accumulation is controversial. Previous pathological studies suggested that the reserve capacity is related to decreased cognitive impairment in the presence of Aβ pathology but not tau pathology. However, a recent study using tau-PET found that a higher intelligence quotient was associated with an attenuated association between tau accumulation and cognitive decline (Halawa et al., 2019). Additional exploration of the cognitive reserve capacity will help identify individuals with a higher tolerance of tau pathologic burden in future studies, and help in the early diagnosis and intervention for AD patients.

If the network efficiency relates to the cognitive and clinical performance, then the FC alteration would predict the neurodegenerative process, particularly for the tau accumulation in hub regions. A negative FC might indicate a connectivity disruption in the temporal lobe and parietal cortex, which leads to an advanced stage of AD.

[^18^F]-THK5117 has shown a high affinity for and selective binding to tau pathology (Harada et al., 2015; Lemoine et al., 2015). Its *S*-form enantiomer [also known as (^18^F)-THK5317] has exhibited favorable pharmacokinetics (Jonasson et al., 2016). It was reported that, except for tau deposition in the neocortex, the monoamine oxidase-B (MAO-B) in the entire brain is correlated with retention of [^18^F]-THK5317 (Harada et al., 2018). However, MAO-B is primarily localized in the inner mitochondrial membrane of astrocytes and linked to the presence of astrogliosis with the accumulation of misfolded proteins. In addition to tau deposition, the activation of microglia and astrogliosis also contributes to the development of AD (Leyns and Holtzman, 2017). Therefore, [^18^F]-THK5317 retention in the AD neocortex is expected to indicate the distribution of tau pathology and reflect the presence of reactive astrocytes *in vivo*.

There are several limitations to this study. First, although all AD participants exhibited positive PIB-PET results, we did not include the influence of Aβ. Schultz et al. (2017) found that tau and Aβ both affect FC, and tau-FC associations were stronger and increased with Aβ levels (Schultz et al., 2017; Adams et al., 2019; Franzmeier et al., 2019). Second, in addition to tau, [^18^F]-THK5317 retention has been reported to reflect reactive astrocytes (Shigemoto et al., 2018). Third, only the MMSE scores were used to evaluate the cognitive level of AD patients. More subdomain evaluations, such as memory, executive function, and others, are needed to assess the cognition of AD patients more accurately. Finally, the cross-sectional nature and small sample size limited our interpretation of causality. Additional longitudinal studies with larger sample sizes are needed to investigate whether tau deposits accurately predict atrophy and decreases in FC.

## Conclusion

Therefore, the cortical regions, including the bilateral inferior lateral temporal lobe, dorsal prefrontal cortex, precuneus, posterior cingulate cortex, hippocampus, and occipital lobe, showed significantly higher [^18^F]-THK5317 accumulation in patients with AD. Decreased FC in regions with higher SUVR was observed in AD patients, and the FC strength negatively correlated with regional SUVR. Patients with a positive FC exhibited older ages, better cognitive performances, and a lower SUVR than patients with a negative FC. The current results indicated that there was an impact of tau deposition on FC at the individual level in AD patients. Furthermore, our findings suggested that the combination of tau-PET and rs-fMRI might be useful to predict the progression of AD.

## Data Availability Statement

The original contributions presented in the study are included in the article/Supplementary Material, further inquiries can be directed to the corresponding author/s.

## Ethics Statement

The studies involving human participants were reviewed and approved by the Chinese PLA General Hospital. The patients/participants provided their written informed consent to participate in this study.

## Author Contributions

LF contributed to the research concept and design, data analysis and interpretation, drafting of the manuscript, critical revision of the article, and final approval of the article. ZZ and LL contributed to data analysis and data interpretation. JZ and XZ contributed to radiosynthesis and data analysis. HX and MZ contributed to data collection and interpretation. RW contributed to the research concept and design. All authors contributed to the article and approved the submitted version.

## Conflict of Interest

The authors declare that the research was conducted in the absence of any commercial or financial relationships that could be construed as a potential conflict of interest.

## Publisher’s Note

All claims expressed in this article are solely those of the authors and do not necessarily represent those of their affiliated organizations, or those of the publisher, the editors and the reviewers. Any product that may be evaluated in this article, or claim that may be made by its manufacturer, is not guaranteed or endorsed by the publisher.

## Footnotes

1
http://www.fil.ion.ucl.ac.uk/spm
