# Supplementary material for: Functional Abnormality Associated With Tau Deposition in Alzheimer’s Disease – A Hybrid Positron Emission Tomography/MRI Study
Source: Front Aging Neurosci. 2021 Oct 13;13:758053. doi: 10.3389/fnagi.2021.758053 (PMC8548365; doi:10.3389/fnagi.2021.758053)
Supplement: Supplementary file 1 [file Table_1.DOCX]

|  | FC (Cluster1 & Cluster2) | FC z score | Tau SUVR | |
| --- | --- | --- | --- | --- |
|  |  |  | Cluster1 | Cluster2 |
| Subject_01 | 0.034861581 | 0.36862144 | 1.1787975 | 1.31524665 |
| Subject_02 | -0.307657964 | -0.723641335 | 1.267949838 | 1.352047277 |
| Subject_03 | 0.235530913 | 0.733268473 | 1.296270953 | 1.376511787 |
| Subject_04 | 0.683958353 | 3.225692874 | 1.216045721 | 1.260486897 |
| Subject_05 | -0.116469005 | -0.175570277 | 1.344897462 | 1.432634859 |
| Subject_06 | -0.030792185 | 0.134540271 | 1.494247782 | 1.616847014 |
| Subject_07 | 0.871610446 | 2.419566506 | 1.308055542 | 1.31979026 |
| Subject_08 | -0.100627756 | -0.253925912 | 1.537346494 | 1.793418091 |
| Subject_09 | 0.545984669 | 2.462921018 | 1.118075107 | 1.216427713 |
| Subject_10 | -0.023949701 | -0.05949693 | 1.679835442 | 1.596554968 |
| Subject_11 | 0.283056116 | 0.594714846 | 1.158477739 | 1.195338782 |
| Subject_12 | -0.455011569 | -1.577660297 | 1.238788124 | 1.436932749 |
| Subject_13 | -0.515981585 | -2.297039672 | 1.35884735 | 1.334552234 |
| Subject_14 | 0.194967583 | 0.991881513 | 1.19940317 | 1.303441899 |
| Subject_15 | -0.868892354 | -1.313692777 | 1.315052644 | 1.43226209 |
| Subject_16 | 0.587114982 | 1.921654044 | 1.063042705 | 1.173781824 |
| Subject_17 | -0.335406456 | -0.853628081 | 1.41003945 | 1.425185912 |
| Subject_18 | 0.348936648 | 1.340544901 | 1.238212283 | 1.426343078 |
| Subject_19 | 0.57071506 | 1.656522086 | 1.445641074 | 1.565204877 |
| Subject_20 | 0.295238376 | 0.649516509 | 1.168659713 | 1.278749934 |
| Subject_21 | 0.476399888 | 1.667213794 | 1.283278126 | 1.289547647 |
| Subject_22 | 0.738956352 | 3.643048483 | 1.268577194 | 1.318775702 |
| Subject_23 | 0.40799067 | 1.481319682 | 1.178977187 | 1.277533155 |
| Subject_24 | -0.569928103 | -1.30354402 | 1.358853328 | 1.511748337 |
| Subject_25 | -0.434948495 | -0.925196607 | 1.436123796 | 1.458204051 |
| Subject_26 | -0.18400625 | -0.631522125 | 1.309875203 | 1.393835893 |

**Supplementary Material 1**

**TABLE 1**. The Value of Functional Connectivity between Cluster 1 and Cluster 2 and SUVR in Alzheimer’s Disease Group

Abbreviations: FC: functional connectivity; SUVR: Standardized Uptake Value Ratio
